# Saline versus balanced solutions: are clinical trials comparing two crystalloid solutions really needed?

**DOI:** 10.1186/s13054-016-1435-x

**Published:** 2016-08-11

**Authors:** Jean-Louis Vincent, Daniel De Backer

**Affiliations:** 1 Department of Intensive Care, Erasme Hospital, Université Libre de Bruxelles, Route de Lennik 808, 1070 Brussels, Belgium; 2 Department of Intensive Care, CHIREC Hospitals, Université Libre de Bruxelles, 1420 Braine l’Alleud, Belgium

Some of our colleagues believe that everything should be tested in prospective, randomized clinical trials. Some of our colleagues also believe that so-called pragmatic trials can help improve the care of critically ill patients. The designs of such studies are based on the postulate that patients are all the same and will be globally either improved or harmed by one strategy compared with another. This may be true for some interventions but is certainly not true for all, including choice of crystalloid solution.

## Saline versus balanced solutions

The difference between saline solutions and balanced solutions is that saline solutions contain 154 mEq/L of sodium and chloride (to be normotonic) and balanced solutions contain anions other than chloride. Hence, liberal administration of saline solutions results in hyperchloremia [1], which can have a number of unwanted effects, including altered intrarenal hemodynamics and perhaps coagulopathy and gastrointestinal symptoms [2, 3]. Without needing to review these effects in detail, one can already see that any deviation of any biological variable from the physiologic range of values will be associated with a worse outcome. Why would this be any different for chloride?

## How do we prescribe fluids?

The choice of intravenous fluid should be based on consideration of certain baseline factors, but also on elements that arise during therapy. For example, at baseline, administration of saline may be indicated in the presence of hypochloremia whereas a hypotonic fluid may be warranted in the presence of hypernatremia. Importantly, the situation may evolve with time as the fluid administered may induce metabolic changes leading to a different “best” fluid choice. Giving several liters of 0.9 % saline will result in hyperchloremia, so continuing this fluid strategy is not rational.

## Let us consider a clinical trial

**Fig. 1:**
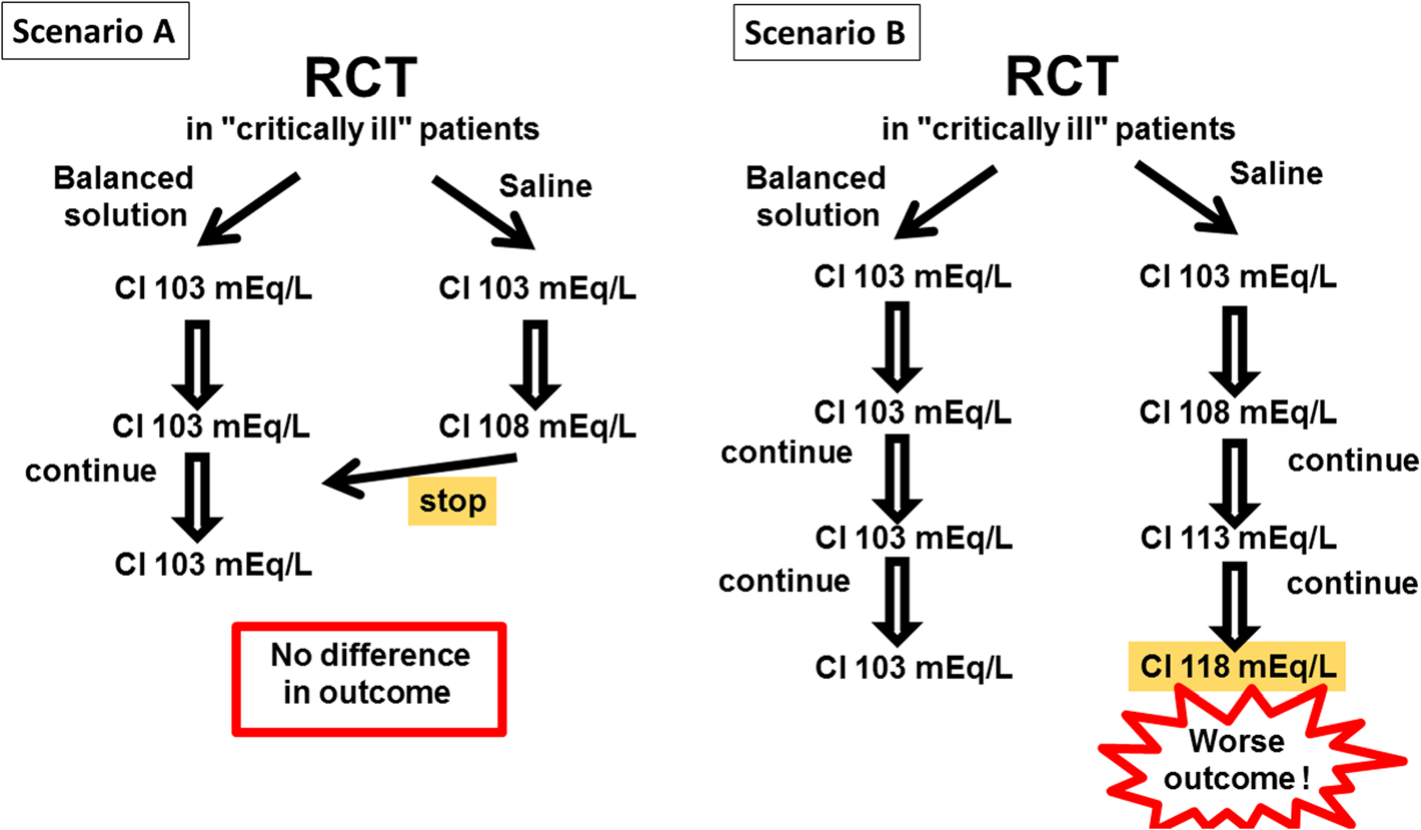
The likely results from a hypothetical randomized clinical trial (*RCT*) comparing a saline solution with a balanced solution in critically ill patients. *Scenario A*: saline solutions are discontinued as soon as hyperchloremia appears, thus preventing the harmful effects of a further increase in chloremia and there will likely be no difference in outcomes in the two arms. *Scenario B*: the administration of saline solutions is continued despite the development of hyperchloremia and the saline group will have worse outcomes than the balanced group

Let us imagine a clinical trial in which saline is compared with a balanced solution (Fig. 1). In the first scenario (panel A), the saline solution is interrupted when hyperchloremia begins to develop. In this scenario, it is unlikely that any harm will be done and the study will conclude that there was no significant difference in outcomes between the two arms. The study will be criticized on the basis that not enough saline was given to the study population to be able to accurately assess the effects of saline. Such an argument has been advanced for the recently completed SPLIT study comparing saline with Plasmalyte [4].

In scenario B, administration of saline solutions is continued, regardless of the test results showing increasing hyperchloremia. Here it is likely that the trial would demonstrate worse outcomes in the saline arm. The overall conclusion would be that saline solutions should be banned or that hyperchloremia should be prevented—these findings could have been predicted without doing the trial! Moreover, the trial would be criticized as it deviates from clinical practice.

Such a study may also have important “negative” clinical and economic consequences. In scenario A, one can argue that, with no differences between the study arms, one should select the cheapest solution and abandon use of costlier, balanced solutions, at least initially. This may result in administration of large amounts of sodium chloride, a condition that was not actually tested in the trial. In scenario B, saline solutions may be banned altogether and only balanced solutions used, thus unduly increasing health care costs. And saline solutions may then no longer be available for specific indications, such as traumatic brain injury (for its normotonicity) or metabolic alkalosis.

## The alternative

We rather propose improved education of the medical community regarding the chemical composition of crystalloid solutions and better understanding of how solutions should be selected depending on each patient’s individual situation. We as clinicians should check blood chloride levels exactly as we do sodium and potassium levels and adapt our fluid choices accordingly.

The real issue for which a fluid trial could be of use is to look at potential beneficial/detrimental effects of added solutes, such as acetate, gluconate, and even calcium or magnesium supplementation, but this would require a particular design.

We should be pleased that we have the choice of several different intravenous solutions, just as we are happy to be able to have a choice of liquids to drink. Any prospective, randomized controlled trial on different types of oral fluid would certainly indicate that excessive amounts of most drinks, especially when they contain alcohol, high sugar, or stimulants, are bad for the body. This does not mean that we should only drink water. But we should vary the types of liquid we drink and drink all liquids in moderate, responsible amounts. Similarly, we should not limit our intravenous fluid choices to just one or two types and should not give any one type in excess.

Undoubtedly, the safest intravenous solution in a patient without major metabolic abnormalities would have a composition close to that of human plasma. It has been said that bicarbonate is unstable when in solution, but this is not true as bicarbonate solutions are readily available from the shelf; actually, renal substitution fluids for continuous hemofiltration contain a mixture of electrolytes, including bicarbonate. Some people already administer these solutions as regular intravenous solutions but the 5-liter bag size complicates this practice. The industry is hesitant to launch such a solution, in anticipation of the costly clinical trials that may be required by the authorities. We argue that a fluid with such a natural composition does not need extensive clinical trials. The scientific community should request that such solutions be developed and insist that the authorities minimize the requirements needed to commercialize such a solution.

There are only a few indications for repeated saline administration: metabolic alkalosis (for the high chloride input) and hyponatremia (for the high sodium input), as well as severe brain injury for its normotonic composition. When such conditions are not present, administration of saline solutions should usually be restricted to not more than 1 liter per 24-h period.

Comparing two intravenous solutions with different compositions makes no sense… unless electrolyte measurements are not available.

## Abbreviations

None.
